# Sub-epidermal Expression of *ENHANCER OF TRIPTYCHON AND CAPRICE1* and Its Role in Root Hair Formation Upon Pi Starvation

**DOI:** 10.3389/fpls.2018.01411

**Published:** 2018-09-27

**Authors:** Louai Rishmawi, Heike Wolff, Andrea Schrader, Martin Hülskamp

**Affiliations:** Botanical Institute, Cluster of Excellence on Plant Sciences (CEPLAS), University of Cologne, Cologne, Germany

**Keywords:** root hair patterning, ETC1, phosphate starvation, expression domains, development

## Abstract

Root hair patterning is best studied in *Arabidopsis thaliana*. A pattern of root hair and non-root hair files is governed by a gene-regulatory network of activators and inhibitors. Under phosphate starvation conditions, extra root hairs are formed in non-root hair positions. This raises the question, whether and how this environmental stimulus is mediated by the known root hair gene network. In this study, we provide genetic and molecular data on the role of *ETC1* in the phosphate starvation induced ectopic root hair formation. We show that the expression in the epidermis is irregular and reduced and that a new expression domain is induced in the sub-epidermis. By expressing *ETC1* in the sub-epidermis, we show that this is sufficient to induce extra root hair formation in N-files. This suggests that the phosphate induced expressional switch from epidermal to epidermal plus sub-epidermal expression of *ETC1* is one environmental input to the underlying patterning network.

## Introduction

In *Arabidopsis thaliana*, the root epidermis exhibits a regular pattern of root hair and non-root hair files. Root hairs develop from files of epidermal cells that are located over the cleft of two underlying cortical cells, whereas epidermal cells that are in contact with only one cortical cell become non-root hair cells (Dolan et al., 1993, 1994; Galway et al., 1994). This pattern is regulated by a complex genetic gene regulatory network that governs the determination of root hair and non-root hair files. The cortical-position dependent regulation of cell fate is mediated by the leucine-rich repeat receptor-like kinase *SCRAMBLED* (*SCM*) (Kwak et al., 2005). *SCM* in turn is regulated by the zinc finger protein JACKDAW (JKD) through a non-cell autonomous signal from the underlying cortical cells (Hassan et al., 2010). SCM negatively regulates the expression of the R2R3 MYB protein WEREWOLF (WER) (Kwak et al., 2005). WER (Lee and Schiefelbein, 1999) acts at an early stage in cell fate determination of the non-root hair cell files by forming an activator complex with the basic helix-loop-helix (bHLH) proteins GLABRA3 (GL3) and ENHANCER OF GLABRA3 (EGL3) (Bernhardt et al., 2003) and the WD40 domain protein TRANSPARENT TESTA GLABRA1 (TTG1) (Walker et al., 1999).

In addition, five homologous R3MYB genes including *CAPRICE* (*CPC*), *TRIPTYCHON* (*TRY*), *ENHANCER OF TRIPTYCHON AND CAPRICE 1, 2*, and *3* (*ETC 1, 2*, and *3*) regulate root hair formation in a redundant manner by suppressing non-root hair formation (Wada et al., 1997; Schellmann et al., 2002; Kirik et al., 2004a,b; Simon et al., 2007; Wester et al., 2009). The respective proteins are considered to compete with WER for binding to GL3 thereby suppressing the activator complex formation (Esch et al., 2003; Ryu et al., 2005; Tominaga et al., 2007). Intercellular interactions are mediated by movement of the R3MYB proteins and GL3. The R3MYB proteins are expressed in the non-root hair cells and move to the hair cells (Wada et al., 2002; Kang et al., 2013). GL3 is expressed in the hair cells and moves to the non-root hair cells (Bernhardt et al., 2005). As a result from these intercellular interactions, the activator complex activates the expression of the homeodomain leucine zipper protein GLABRA2 (GL2) in non-hair cells, where it represses root hair cell fate (Galway et al., 1994; Hung et al., 1998). Here, GL2 negatively regulates the expression of down stream genes. One of the down-stream genes is the basic helix loop helix gene *ROOT HAIR DEFECTIVE 6* (*RHD6*) (Menand et al., 2007). *RHD6* is essential for root hair initiation and also one of its direct targets *ROOT HAIR DEFECTIVE 6-LIKE 4* (*RSL4*) is expressed in the root hair cells where it acts as a regulator of root hair growth (Yi et al., 2010).

Under Pi deficient growth conditions, *A. thaliana* plants produce higher density of root hairs (Bates and Lynch, 1996; Ma et al., 2001; Muller and Schmidt, 2004). This increase in root hair density can be explained by the decrease of the longitudinal length of epidermal cells though. However, the decrease in the epidermal cell length in *wer, scm* and *cpc* mutants under Pi starvation conditions did not account for the increase of root hair cells, pointing toward an additional mechanism triggering extra root hair formation (Savage et al., 2013). A role in the determination of root hair and non-root hair fate was suggested for *BHLH32* (Chen et al., 2007). Mutations in *BHLH32* lead to an increase of root hairs under Pi-starvation. It was suggested that BHLH32 regulates root hair patterning under Pi starvation through direct interaction with GL3 and TTG1 (Chen et al., 2007).

In the present study, we investigate the role of the root hair patterning machinery under phosphate-starvation. We show that in the absence of GL2, WER, and TTG1 phosphate starvation cannot trigger additional root hair formation. We further show that ETC1 plays a role in the increase of root hair number under Pi- conditions. We report that ETC1 expression changes such that it is irregular in the epidermis and turned on in the sub-epidermis. We provide evidence that ETC1 can move from the sub-epidermis and that this is sufficient to induce root hair formation.

## Results and Discussion

### Phosphate Starvation Promotes Root Hair Formation Through the Patterning Machinery

In order to study the possible role of genes controlling root hair pattering in the phosphate starvation response, we compared the formation of root hairs in root hair (H-position) and non-root hair (N-position) files between wild type and mutants. While previous studies used older plants, we aimed to capture in particular early events of pattern formation by analyzing root hair patterns on 7-day old seedlings grown either on Pi+ or Pi- conditions (**Supplementary Table S1**). We reasoned that this might reveal different results as in experiments done under low phosphate levels [e.g., *cpc-1* (Ws), *try-29760* (Col-0), and *etc1-1* (Col-0) (Chen and Schmidt, 2015)] or phosphate shift experiments (Muller and Schmidt, 2004; Savage et al., 2013; **Supplementary Table S1**). In this study, we grew seedlings under long day conditions on agar plates. We analyzed root hair formation in H and N positions in *gl2-1* (L*er*), *wer-1* (Col-0), *ttg1-1* (L*er*), *cpc-2* (Col-0), *try-JC* (Col-0), *etc1-1*, and *cpc-2 etc1-1* mutants. Due to high plasticity of quantitative root hair phenotypes we present the results of two independent experiments and consider only results that are statistically significant in both experiments.

**FIGURE 1:**
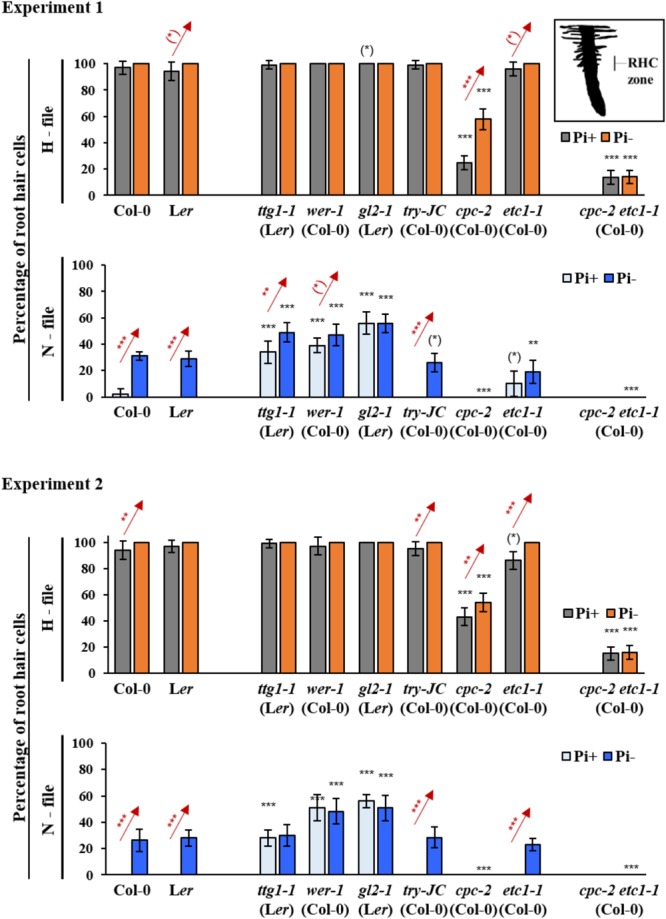
Quantitative analysis of root hair formation in response to phosphate starvation in wild type and mutant genotypes. Two independent experiments are shown in which root hair number is compared in N- and H files between at least 10 wild type and mutant seedlings under normal conditions and under phosphate starvation conditions. RHC zone indicates the region of root hair analysis. Red stars indicate a significant difference between Pi+ and Pi– conditions at ^∗^*p* < 0.5, ^∗∗^*p* < 0.01, ^∗∗∗^*p* < 0.001 (Wilcoxon test). Black stars indicate a significant difference between wild type and mutants at ^∗^*p* < 0.5, ^∗∗^*p* < 0.01, ^∗∗∗^*p* < 0.001 (Wilcoxon test).

As described previously, both wild-type ecotypes L*er* and Col-0 produced extra root hairs in non-root hair cells when grown under Pi- conditions (**Figure 1** and **Supplementary Tables S2, S3**) (Muller and Schmidt, 2004). As expected, we found ectopic root hairs in *gl2-1, wer-1*, and *ttg1-1* mutants. In contrast to a previous study (Muller and Schmidt, 2004), we found no statistically significant difference of ectopic hairs in non-root hair files under Pi- conditions in these three mutants (**Figure 1** and **Supplementary Tables S2, S3**). This suggests that they are required for Pi- induced extra root hair formation under our growth conditions. The analysis of the R3MYB mutants *cpc-2, try-JC*, and *etc1-1* revealed different responses. In contrast to Chen and Schmidt (2015), root hair formation in *try-JC* mutants was indistinguishable from wild type under Pi+ and Pi-conditions (**Figure 1** and **Supplementary Tables S2, S4**). *cpc-2* mutants have a reduced number of root hairs in root hair positions that is strongly increased under Pi-conditions (**Figure 1** and **Supplementary Tables S2, S3**) see also (Muller and Schmidt, 2004; Chen and Schmidt, 2015). Root hair number in *etc1-1* mutants was similar to wild-type under Pi+ conditions (Kirik et al., 2004a). For the *etc1-1* mutant, a slight decrease of root hair numbers in N-files under Pi- conditions was reported by others (Chen and Schmidt, 2015). We also observed a small decrease in one of our two sets of experiments (**Figure 1** and **Supplementary Tables S2–S4**). Given that the reduction of root hairs is very low (in the 10% range) in the report by (Chen and Schmidt, 2015) as well as in our experiment it is conceivable that such a low deviation may be difficult to detect in different experiments.

We also analyzed the *cpc-2 etc1-1* double mutant under Pi+ and Pi- conditions. Similar to previous studies (Savage et al., 2013; Chen and Schmidt, 2015), the *cpc-2 etc1-1* double mutant did not produce ectopic root hairs under Pi- conditions suggesting no independent effect of *ETC1* (**Figure 1** and **Supplementary Tables S2, S3**). However, an *ETC1* dependent response in root hair production in H files is evident when comparing the *cpc-2* single mutant with the *cpc-2 etc1-1* double mutant. While the *cpc-2* mutant shows a higher number of root hairs in H files under phosphate starvation conditions, the double mutant does not respond. These results support the idea that *ETC1* is involved in the regulation of root hair patterning under Pi- conditions.

### Spatial Expression of Root Hair Patterning Genes Under Pi- Conditions

**FIGURE 2:**
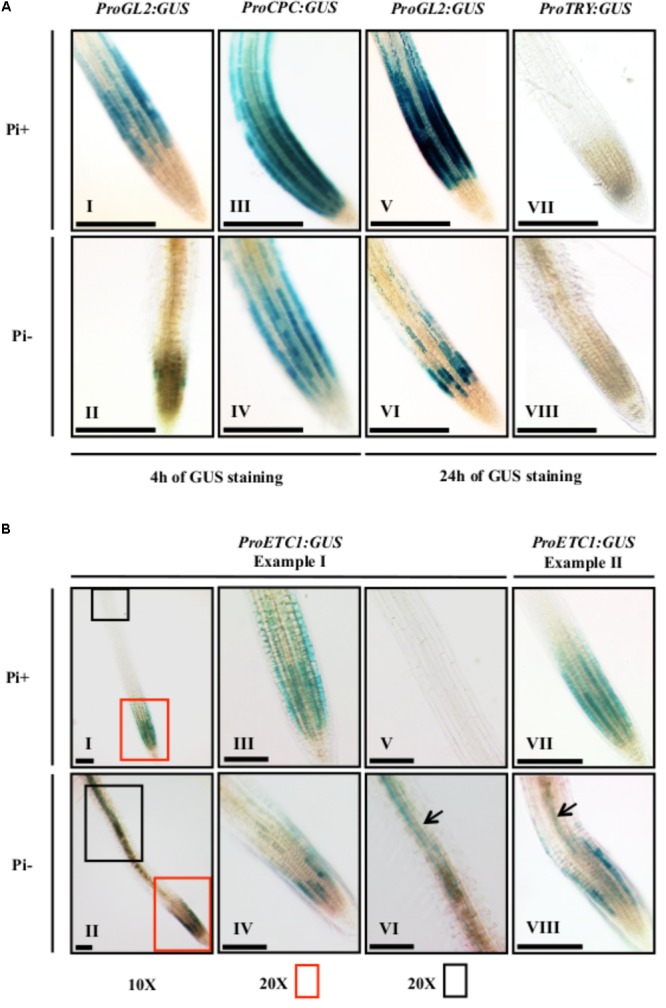
Histochemical GUS expression analysis of Pro*CPC*, Pro*GL2*, Pro*TRY*, and Pro*ETC1.* GUS expression staining was done on 7-day old seedlings grown on Pi+ media and Pi– media. **(A)** GUS expression of Pro*CPC, ProGL2*, and Pro*TRY.* GUS staining was done for 4 h in seedlings (I–IV) and for 24 h in seedlings (V–VIII). A reduction in GUS expression was noticed for Pro*GL2* and Pro*CPC* in seedlings grown under Pi– conditions (II, IV, VI) as compared to seedlings grown on Pi+ (I, III, V). No GUS expression was detected for Pro*TRY* under both Pi+ and Pi– conditions (VII, VIII). **(B)** *GUS* expression of Pro*ETC1*. Two examples are shown to depict the variability of the expression pattern. Plant I (I, II) shows an overview and higher magnifications of the root tip (III, IV) and a more distal region (V, VI). A new expression domain of Pro*ETC1* was found in the sub-epidermis under Pi– conditions (black arrows in VI and VIII). Scale bars: 200 μm.

Several studies had shown that the expression levels of root hair patterning genes were altered under phosphate starvation (Wu et al., 2003; Misson et al., 2005; Lan et al., 2012; Chen and Schmidt, 2015). For *ETC1* expression, conflicting results were obtained. Two studies reported an up-regulation of *ETC1* expression (Misson et al., 2005; Lan et al., 2012) whereas no differences were detected in the study of Chen and Schmidt (2015). As for patterning processes expression levels are not necessarily relevant but rather the correct spatial expression matters, we decided to assess the spatial expression of *CPC, GL2, TRY*, and *ETC1* under Pi+ and Pi- conditions using established promoter:GUS marker lines. To judge changes in the relative expression levels we monitored the GUS staining after 4 and 16 h. In agreement with other studies, the expression of *pGL2*:*GUS* was found in non-root hair files under normal conditions (**Figures 2A** – I, V) (Masucci and Schiefelbein, 1996). After 4 h of staining, we found almost no GUS staining in Pi- treated roots (**Figure 2A** – II) and after 16 h we observed a discontinuous staining in non-root hair files (**Figure 2A** – VI). This is consistent with the formation of extra root hairs in approximately 30% of the cells under Pi- conditions (**Supplementary Table S2**). As compared to normal growth conditions (**Figure 2A** – III; Lee and Schiefelbein, 2002) *pCPC*:*GUS* expression levels were clearly reduced and discontinuous in N-files in Pi- media grown seedlings after 4 h of GUS staining (**Figure 2A** – IV). *pTRY*:*GUS* was neither detected under normal conditions (Schellmann et al., 2002) nor in Pi- grown seedlings after 16 h GUS staining (**Figures 2A** – VII, VIII). Expression of *pETC1*:*GUS* was previously reported to be in N-files (Kirik et al., 2004a) which was confirmed in *in situ* hybridization experiments (Simon et al., 2007). Consistent with this we found *pETC1*:*GUS* expression in cell files under Pi+ conditions (**Figures 2B** – I, III, VII). Under Pi- conditions, the epidermal expression of ETC1 was irregular such that large regions including N-files showed almost no GUS staining and some files were normally stained (**Figures 2B** – II, IV, VIII). An additional expression domain was observed in sub-epidermal cells (**Figures 2B** – VI, VIII) which was never noticed under Pi+ conditions (**Figure 2B** – V).

### Promoter Analysis of ETC1

**FIGURE 3:**
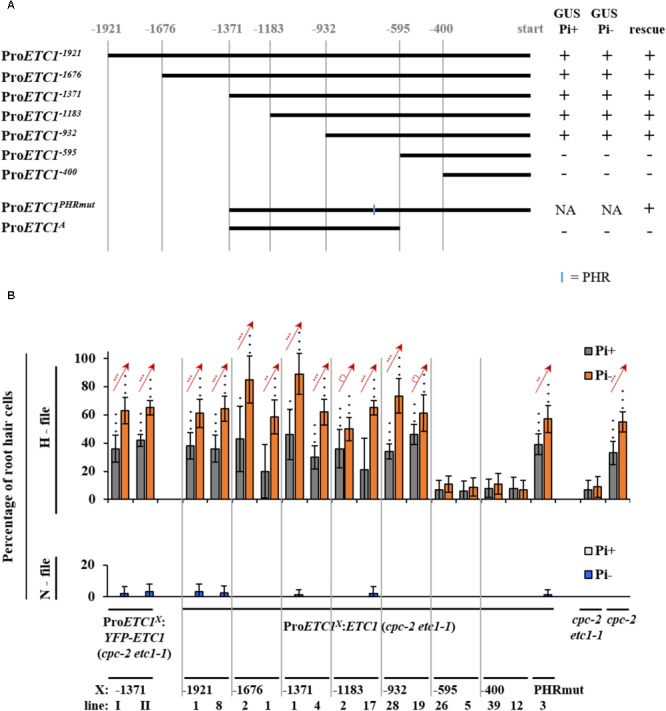
Promoter deletion analysis of *ETC1*. **(A)** Schematic diagram showing a serial deletion of the 5′ regulatory region of *ETC1*. Vertical lines show the relative position of the fragment with respect to the *ETC1* start codon. The blue line indicates the location of the phosphate response binding (*PHR-1*) site. **(B)** Rescue analysis of the ETC1 promoter fragments in at least ten plants. Red stars indicate a significant difference between Pi+ and Pi– conditions at ^∗^*p* < 0.5, ^∗∗^*p* < 0.01, ^∗∗∗^*p* < 0.001 (Wilcoxon test). Black stars indicate a significant difference between wild type and mutants at ^∗^*p* < 0.5, ^∗∗^*p* < 0.01, ^∗∗∗^*p* < 0.001 (Wilcoxon test). + Indicates that the construct was able to rescue or was able to drive GUS expression, – indicates that the construct was not able to rescue or was not able to drive GUS expression.

The expression analysis of the *ProETC1*:*GUS* line indicates an expression switch from regular epidermal expression in N-files to subepidermal expression upon phosphate starvation. In an attempt to dissect these regulation events, we created a series of deletion constructs driving either the expression of the GUS-marker gene or the ETC1 CDS for rescue experiments (**Figure 3A**). As the *etc1-1* single mutant root hair phenotype is fairly subtle and variable, we used the *cpc-2 etc1-1* double mutant for rescue experiments. A 932 bp long promoter fragment was sufficient to mediate rescue of the *cpc-2 etc1-1* mutant phenotype to a level found in the *cpc-2* mutant and normal expression patterns (**Figures 3A,B** and **Supplementary Tables S5–S9**).

**FIGURE 4:**
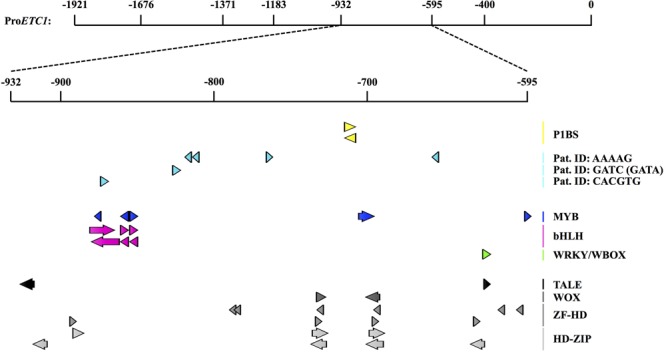
*Cis*-regulatory elements and motifs related to Pi responsiveness and patterning with the selected ProETC1 fragment. Detailed analysis of *cis*-regulatory motives in the Pro*ETC1^-932- -595^* fragment using PlantPAN 2.0 (Chow et al., 2016) and manual analysis with motifs from a recent publication (Salazar-Henao et al., 2016). The figure was constructed using the CLC DNA Workbench (see text footnote 4). P1BS was previously identified by PLACE (Higo et al., 1999) mutated in this study and did not show to be causative. Recently, 14 root specific Pi-responsive motifs were identified (Salazar-Henao et al., 2016). Three of these were found in the selected Pro*ETC1* fragment (labeled “Pat. ID”). Below, patterning related motifs extracted from a PlantPAN 2.0 analysis are shown. Orientation of motifs: +: arrows point to the right, – strand: arrow points to the left. P1BS, PHR1-binding site; Pat. ID, pattern identifier from Salazar-Henao et al. (2016); TALE, three amino acid loop extension (of homeodomains); WOX, WUS homeobox-containing; ZF-HD, zinc-finger-homeodomain; HD-ZIP, homeodomain-leucine zipper. All results and precise positions can be found in **Supplementary Table S11**.

As all Pro*ETC1* fragments were able to rescue *cpc-2 etc1-1* mutant phenotype except for Pro*ETC1^-595^* and Pro*ETC1^-400^*, we expected the existence of regulatory sequence between -932 and -595 region to be essential for the expression of *ETC1*. At this time point we used the PLACE tool to search for MYB and phosphate response (PHR-1) binding sites https://sogo.dna.affrc.go.jp/cgi-bin/sogo.cgi?lang=en&pj=640&action=page&page=newplace (Higo et al., 1999) (**Supplementary Table S10**). We found one PHR-1 binding site, however, changing its sequence from (gtatatcc) to (aaaaaaaa) did not alter the promoter rescuing ability to *cpc-2 etc1-1* double mutant under phosphate starvation conditions (**Figure 3B** and **Supplementary Tables S5–S9**). This suggests the presence of other phosphate regulatory elements that control the *ETC1* expression based on phosphate availability. We therefore analyzed the relevant promoter region using more recent tools and data sets. Toward this end we focused on 14 *cis*-regulatory elements from co-expression-derived modules that are involved in the root hair formation induced by phosphate deficiency (Salazar-Henao et al., 2016). Three of these *cis*-regulatory elements are present in the relevant ProETC1 fragment (**Figure 4** and **Supplementary Table S11**): the AAAAG pattern, regulated by Dof (DNA-binding with one finger) transcription factors, the GATC pattern, regulated by GATA transcription factors and the CACGTG pattern, a G-box which occurs in phytochrome A-responsive promoters (Salazar-Henao et al., 2016). The CACGTG motif is of particular interest as both bHLH/MYB and other factors can potentially bind to this region. Thus this region might play a role to coordinate developmental regulation and the Pi starvation response.

### Spatial Distribution of YFP-ETC1 Protein

**FIGURE 5:**
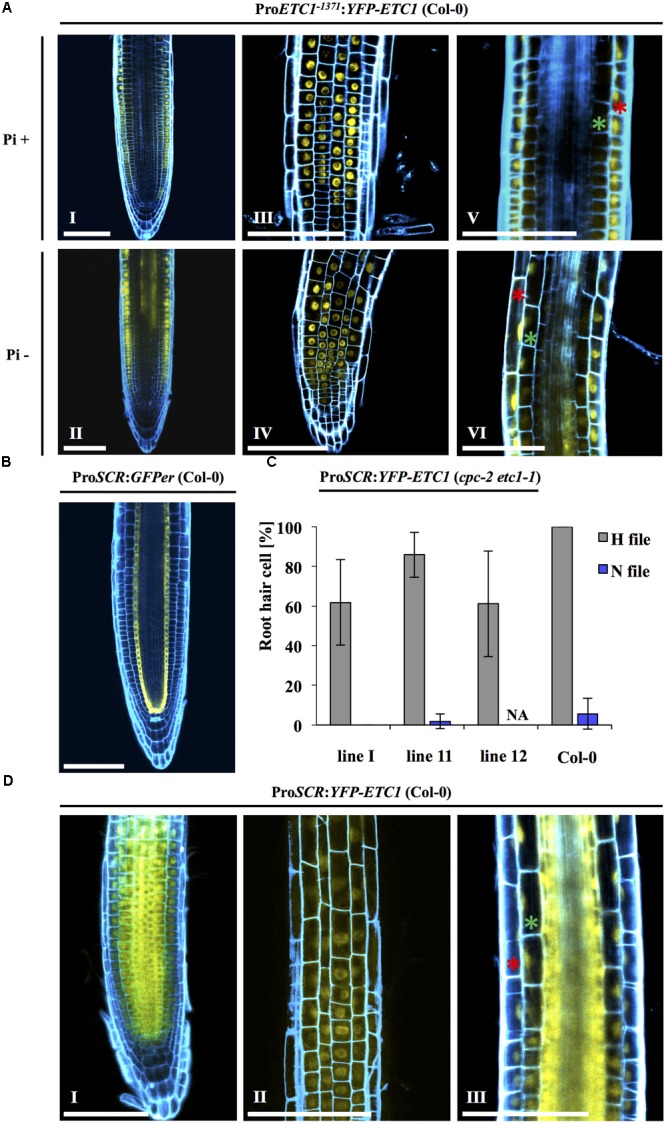
Intercellular motility and localization of YFP-ETC1 under the *ETC1* and *SCR* promoters. **(A)** Root of a *cpc-2 etc1-1* double mutant plant carrying Pro*ETC1*:*YFP-ETC1* under Pi+ and Pi– conditions. Under Pi+ conditions, YFP-ETC1 is seen in the root hair and non-root hair file epidermal cells (I, III) but not in the ground tissue (V). Under Pi– conditions, YFP-ETC1 was observed in the root hair and non-root hair file epidermal cells (II, IV) and in the ground tissue (VI). (I, II) Pictures show overview, (III, IV) pictures show focal planes of the epidermis and (V, VI) pictures show the focal planes in the cortical cells and the stele. Red stars mark epidermal cells, green stars mark cortical cells. Scale bar = 100 μm. **(B)** CLSM pictures of roots from transgenic Pro*SCR:GFPer* (Col-0) plants grown under Pi+ conditions. Blue: propidium iodide, yellow: YFP. **(C)** A diagram showing the rescuing ability of Pro*SCR:YFP-ETC1* in the *cpc-2 etc1-1* double mutant under Pi+ conditions. **(D)** Pro*SCR:YFP-ETC1* (Pi+ conditions). (I) Overview shows YFP-ETC1 in all tissues. (II) Focal plane showing YFP-ETC1 in epidermal cells. (III) Focal plane showing YFP-ETC1 in the epidermal cells and the ground tissue. Red star labels an epidermal cell, green star labels a cortical cell. Scale bar = 100 μm.

The reduced expression of ETC1 in the epidermis is not consistent with a role of *ETC1* in increased root hair formation under Pi- conditions. However, it is conceivable that ETC1 protein can move from the sub-epidermal cells and contributes to the regulation of epidermal cell fate. In this scenario, ETC1 might be expected to be transported equally well to N and H files. To explore this question, we established plants carrying the Pro*ETC1*:*YFP*-*ETC1* construct. In a first step, we demonstrated that the promoter and the fusion proteins are fully functional by rescuing the *cpc-2 etc1-1* double mutant (**Figure 3B** and **Supplementary Table S6**). Pro*ETC1*:*YFP*-*ETC1 cpc-2 etc1-1* plants showed a root hair phenotype similar to *cpc-2* mutant under Pi+ and Pi- conditions (**Supplementary Table S6**). Under Pi+ conditions, YFP-ETC1 protein was found in N- and H- files (**Figure 5A**). Thus, similar as shown for CPC (Wada et al., 2002), *ETC1* is expressed in N-files and moves into the H files. In Pro*ETC1*:*YFP*-*ETC1* plants grown under Pi-conditions, the YFP-ETC1 signal was observed in all root layers with elevated levels in the stele and epidermal cells (**Figures 5A** – II, IV, VI). When comparing the GUS expression with the YFP-ETC1 distribution we noted clear differences. In particular, the region more distal from the root tip displaying ETC1 expression in the sub-epidermis clearly exhibited YFP-ETC1 protein in the epidermis (**Figures 2B** – VI, **5A** – II). This suggests that YFP-ETC1 RNA or protein can (**Figure 5A** – II) move between cell layers. We also noted that the pattern irregularities found for the GUS expression is much less pronounced for the YFP-ETC1 signal. This is likely to be due to YFP-ETC1 mobility that levels out expression differences between cells.

To test whether movement of ETC1 from the subepidermis to the epidermis can regulate root hair patterning, we expressed the *ETC1* CDS under the *SCARECROW* (*SCR*) promoter in the *cpc-2 ect1-1* double mutant and analyzed the resulting plants for root hair rescue under Pi+ conditions. As expected, Pro*SCR:GFPer* (**Figure 5B**) showed expression in the cortical/endodermis initial cells and in the endodermis (Helariutta et al., 2000). In Pro*SCR*:*YFP-ETC1* plants, YFP-ETC1 was detected in all cell layers indicating that YFP-ETC1 RNA or protein can move between several layers (**Figure 5D**). This is consistent with the report from Rim et al. (2011). In a systematic approach it was shown that transcription factors may be cell-autonomous, may move several cell layers or to all layers including the epidermis (Rim et al., 2011). Interestingly, CPC was shown to belong to the latter class supporting our finding that ETC1 can move through all cell layers.

The phenotypic analysis revealed, that the Pro*SCR*:*YFP-ETC1 cpc-2 etc1-1* plants exhibited more root hairs in H files than the *cpc-2* single mutant (**Figure 5C** and **Supplementary Table S12**) indicating that expression of ETC1 in the stele is sufficient to rescue the mutant phenotype. It is therefore conceivable that the expression switch of ETC1 upon phosphate starvation from N-files to the sub-epidermis is functionally relevant and that it contributes to the ectopic root hair formation.

## Conclusion and Outlook

Despite the functional redundancy of *ETC1* with other R3 MYB genes such as *CPC, ETC3*, and *TRY* under normal growth conditions it is interesting that they appear to have divergent functions under Pi starvation conditions as noted before (Chen and Schmidt, 2015). The four paralogous genes strikingly differ with respect to changes in RNA abundance and root hair formation upon Pi- starvation (Chen and Schmidt, 2015). Among them, *ETC1* appears to have a less prominent role as the number of root hairs is only weakly effected by Pi starvation (Chen and Schmidt, 2015). Only the comparison of *cpc* and the *cpc etc1* double mutant unambiguously revealed a function of *ETC1* in Pi starvation responses. However, the mechanism by which ETC1 contributes to Pi starvation response is novel and a potentially powerful principle to override other regulation events. The Pi starvation induced new expression domain in subepidermal cell layers in combination with the fact that ETC1 can travel through several cell layers represents a developmental switch by which a root hair promoting factor can be provided in all epidermal cells and thereby promoting root hair formation.

## Materials and Methods

### Plant Material, Culture Media, and Root Hair Analysis

The following *A. thaliana* lines were used in this study: Columbia (Col-0), Landsberg *erecta* (L*er*), *ttg1-1* (L*er*) (Koornneef, 1981), *gl2-1* (L*er*) (Di Cristina et al., 1996), *wer-1* (Col-0) (Lee and Schiefelbein, 1999), *cpc-2* (Col-0) (Kurata et al., 2005), *try-JC* (Col-0) (Larkin et al., 1999) and *etc1-1* (Kirik et al., 2004a), *pGL2:GUS, pCPC:GUS* (Col-0) (Pesch et al., 2013), *pTRY*:*GUS* (Col-0) (Pesch and Hulskamp, 2011) and *pSCR*:*GFPer* (Col-0) (Rishmawi et al., 2014). *pETC1*:*GUS* (Col-0), *pETC1*:*YFP*-*ETC1* (Col-0), and *pSCR*:*YFP*-*ETC1* (Col-0) were created in this study as described below. The *cpc-2 etc1-1* double mutant was created by crossing *cpc-2* with *etc1-1*.

Seeds were sterilized by shaking in 100% ethanol followed by adding 4% HCL, washing three times with sterile water. After 3 days of vernalization at 4°C seedlings were grown on vertically placed MS (Murashige and Skoog, 1962) agar plates with (Pi+) and without (Pi-) phosphate under long day conditions (16 h light/8 h dark) for 7 days. Pi+ and Pi- MS medium contained 2.06 mM NH_4_NO_3_, 1.88 mM KNO_3_, 0.31 mM MgSO_4_, 0.1 mM MnSO_4_, 0.03 mM ZnSO_4_, 0.1 mM CuSO_4_, 0.3 mM CaCl_2_, 5.0 mM KI, 0.1 mM CoCl_2_, 0.1 mM FeSO_4_, 0.1 mM EDTA, 0.1 mM H_3_BO_3_, and 1 mM Na_2_MoO_4_.2H_2_O supplemented with 1% (w/v) agar and 1% (w/v) sucrose. 1 mM KH_2_PO_4_ was added for MS Pi+ and replaced by 1 mM of KCL for MS Pi-. The pH was adjusted to 5.6–5.8 by adding NaOH.

For root hair analysis epidermal files were defined by their relative position to the cortical cells. At least 10 biological replicas were analyzed for each genotype. For each seedling, the number of root hairs was determined for 10 cells in the H-file and 10 cells in the N-position.

### Plasmid Construction

For Pro*ETC1*:*GUS* constructs, serial deletions were created as presented in **Figure 3**. For all the constructs, genomic DNA of Col-0 and primers with attb sites were used: GW-ProETC1 F and GW-ProETC1 R primers for Pro*ETC1*^-1921^, GW-ProETC1^-1676^ F and GW-ProETC1 R primers for Pro*ETC1*^-1676^, GW-ProETC1^-1371^ F and GW-ProETC1 R primers for Pro*ETC1*^-1371^, GW-ProETC1^-1183^ F and GW-ProETC1 R primers for Pro*ETC1*^-1183^, GW-ProETC1^-932^ F and GW-ProETC1 R primers for Pro*ETC1*^-932^, GW-ProETC1^-595^ F and GW-ProETC1 R primers for Pro*ETC1*^-595^, GW-ProETC1^-400^ F and GW-ProETC1 R primers for Pro*ETC1*^-400^. Each of the produced fragments was introduced in pDONR201 entry vector. The entry vectors were used to transfer the *ETC1* fragments to the destination vector (ProBat-TL-B-C-GUS-FUS-w/oPromoter) through LR reactions (Clontech).

ProBat-B-C-*GUS-FUS*-w/oPromotor was generated as follows. ProBat-B-*sYFPN*-w/oPromoter (provided by Cordula Jörgens) contains unique restriction sites replacing Pro35S-TL upstream of a Gateway^TM^ (Invitrogen^1^) cassette in ProBat-TL-B-*sYFPN* (J.F. Uhrig, unpublished data). *sYFPN* was removed (SpeI) to generate ProBat-B-w/oPromotor. *GUS-FUS* with attached SpeI sites was amplified with ANS89SpeI-HA-GUS-s and ANS90SpeI-GUS-as (**Supplementary Table S12**) from an entry vector (Rudolph et al., 2003) and inserted into the SpeI site following the Gateway^TM^ cassette of ProBat-B-w/oPromotor to obtain ProBat-B-C-*GUS-FUS*-w/oPromotor.

For the rescuing experiments with ProETC1 fragments *ETC1* CDS was amplified from cDNA using the GW-ETC1 cDNA F and GW-ETC1 CDS R primers (**Supplementary Table S13**) and introduced in *pDONR201*. The *ETC1* CDS was introduced in the Pro*Bat-B-w/oPromotor* destination vector by LR reaction (Clontech). Each fragment of Pro*ETC1* was introduced *in* Pro*Bat-B-w/oPromotor-ETC1* CDS by blunt ending cloning using the Smi1 restriction enzyme.

For *pENSG-GW-*Pro*ETC1:YFP-ETC1*: The *ETC1* CDS was introduced in *pENSG-YFP* destination vector (Wester et al., 2009) by LR reaction (Clontech). The *ETC1* promoter (1371 bp upstream of start codon) was amplified using Asc1-pETC1 F and Xho1-pETC1 R primers (**Supplementary Table S13**) and introduced in pJET1.2 (Fermentas, United States). Finally Pro*ETC1* was cloned in the *pENSG-YFP-ETC1* destination vector as an Asc1 and Xho1 fragment.

For *pENSG-GW-*Pro*SCR:YFP-ETC1* was created analogous as described for *pENSG-GW-*Pro*ETC1:YFP-ETC1* using a Pro*SCR* CDS amplified using Asc1-pSCR F and Xho1-pSCR R primers (**Supplementary Table S13**).

*pENSG-GW-*Pro*ETC1^PHR1mut^:YFP-ETC1* the ETC1 CDS was introduced by LR reaction as previously described. Pro*ETC1^PHR1mut^* promoter was amplified in three steps. The first fragment was amplified using Asc1-Pro*ETC1* F and phr1mut- R primers. The second fragment was amplified using Xho1-Pro*ETC1* R and phr1mut- F primers. These two fragments were used as a template for the third PCR using Asc1-pETC1 F and Xho1-pETC1 R primers (**Supplementary Table S13**). The phr1mut-F and phr1mut-R primers contain the substituted nucleotides that will convert the PHR-1 binding sequence from (gtatatcc) to (aaaaaaaa). The Pro*ETC1^PHR1mut^* was cloned in the Pro*ENSG-YFP-ETC1* destination vector as an Asc1 and Xho1 fragment.

### GUS Staining

GUS staining was done with 7-day old seedlings as described previously (Vroemen et al., 1996). Seedlings were fixed (50% methanol; 10% acetic acid) at 4°C for 24 h, transferred to 80% ethanol for 2 h, washed three times with water and analyzed by transmission light microscopy.

### Microscopy and Image Acquisition

Pictures of GUS stained roots were acquired using a Leica DMRA microscope (Leica Microsystems) equipped with DISKUS software (Carl H. Hilgers-Technisches Büro).

Confocal laser-scanning microscopy (CLSM) was performed using a Leica TCS-SP8 confocal microscope equipped with the Leica LAS AF software. For imaging a Leica 1.2 NA, 63× water objective was used. The propidium iodide staining was excited using a DPSS laser at 561 nm, the GFP/YFP signal was excited using an argon laser at 488/514 nm. For the protein localization, seedlings were stained with 100 μg/ml of propidium iodide for 1 min followed by washing the samples with water. The analyses were done by sequential scanning starting with the respective higher wavelength. CLSM pictures (focal planes) of each channel were merged in Fiji (Schindelin et al., 2012) and the colors were set as follows: “Cyan Hot” for propidium iodide and “Yellow” for YFP. For each channel, only brightness and contrast were additionally adjusted.

### Statistical Analysis and Motif Analysis

Statistical analyses were done with R using RStudio (R Core Team, 2017). Normality was tested for with the Kolmogorov–Smirnov test. As data for almost all genotypes and conditions were not normally distributed, an unpaired two-samples Wilcoxon test was conducted.

The motif analysis was conducted using PlantPAN 2.0^2^ (Chow et al., 2016) and manual analysis with 14 *cis*-regulatory elements related to the formation of phosphate deficiency-induced root hairs before (Salazar-Henao et al., 2016). The figure was constructed using the CLC DNA Workbench^3^.

## Author Contributions

LR did the most experiments. HW did the confocal image acquisition and root hair phenotyping analysis of selected lines. LR, MH, and AS conceived the study and wrote the manuscript. AS and LR analyzed the data. AS did the statistics and motif analysis. All authors have approved the final article.

## Conflict of Interest Statement

The authors declare that the research was conducted in the absence of any commercial or financial relationships that could be construed as a potential conflict of interest.

## Footnotes

1 http://www.thermofisher.com/de/de/home/brands/invitrogen.html

2 http://plantpan2.itps.ncku.edu.tw/

3 www.clcbio.com
