# Supplementary material for: Sub-epidermal Expression of ENHANCER OF TRIPTYCHON AND CAPRICE1 and Its Role in Root Hair Formation Upon Pi Starvation
Source: Front Plant Sci. 2018 Sep 27;9:1411. doi: 10.3389/fpls.2018.01411 (PMC6171471; doi:10.3389/fpls.2018.01411)
Supplement: Supplementary file 1 [file Table_1.docx]

**Table S1:** Summary of the used mutants, methods for root hair analysis and growth conditions among different studies

| **Source** | **mutants** | **method** | **Growth conditions** |
| --- | --- | --- | --- |
| Chen and Schmidt 2015 | *cpc-1 (Ws)*, *try-29760* (Col-0), ***etc1-1* (Col-0)** | Root hair density was measured from 2 to 6 mm from the tip of the primary root | Low phosphate conditions. Medium contained 2.5 μM KH2PO4 and gelrite.  Light: continuous illumination |
| Savage et al., 2013 | *cpc (?)*, *wer(?)*, ***etc1-1* (Col-0),** *scm*, *cpc etc1* | number of root hairs was examined in 45 to 50 cross-sections from 10 roots for each genotype | Normal conditions with Phytagel for 10 days, then transferred to agar plates without phosphate for 6 days. Light: continuous illumination |
| Müller and Schmidt 2004 | *gl2?,* *wer (?),* *ttg1* (*ttg* or *ttg-w* (both L*er*)), *cpc (?), erh1, erh3, rhl1, rhl2, rhl3, rhd6, rhd1, trh1, lrx, tip1-2, kjk, rhd2, rhd3, rhd4* | Root hair patterns were analysed in cross sections of 10 primary root apical segments per genotype and treatment. | Normal conditions with agar for 11 days, and then transferred to agar plates without phosphate for 9 days. Light: continuous illumination |
| This study | ***gl2-1* (L*er*)*, cpc-2* (Col-0), *etc1-1* (Col-0), *wer-1* (Col-0), *ttg1-1* (L*er*)*, try-JC* (Col-0), *cpc-2 etc1-1*** | The number of root hairs was determined for 10 cells in the H-file and 10 cells in the N-position. 10-15 seedlings for each genotype. Two biological replicates. | Vertically placed MS agar plates with (Pi+) and without (Pi-) phosphate for 7 days. Light: long day conditions |

Bold: Mutants used in this study.
