# Supplementary material for: Sub-epidermal Expression of ENHANCER OF TRIPTYCHON AND CAPRICE1 and Its Role in Root Hair Formation Upon Pi Starvation
Source: Front Plant Sci. 2018 Sep 27;9:1411. doi: 10.3389/fpls.2018.01411 (PMC6171471; doi:10.3389/fpls.2018.01411)
Supplement: Supplementary file 2 [file Table_2.docx]

**Table** **S2. Effect of Pi availability on root hair patterning.**

Percentage of root hair cells in each cell file in mutants and wild types in the RHC (root hair counting) zone. Two separate experiments are shown. 7-day old seedlings grown under Pi sufficient and deficient conditions were analyzed. Values represent the mean percentages of root hair cells for 10 cells per seedling in at least 10 plants (mean percentage ± SD). See Table S4 and S5 for the results of the statistical analysis.

|  |  | **Root hair cell [%]** | | | | | | |
| --- | --- | --- | --- | --- | --- | --- | --- | --- |
|  |  | **Phosphate sufficient (Pi+)** | | | **Phosphate deficient (Pi-)** | | |  |
| **Genotype** | **Exp.** | **n** | **H-file** | **N-file** | **n** | **H-file** | **N-file** |  |
| **Col-0** | 1 | 10 | 97.0 ± 4.8 | 2.0 ± 4.2 | 10 | 100.0 ± 0.0 | 31.0 ± 3.2 |  |
| **L*er*** | 1 | 10 | 94.0 ± 7.0 | 0.0 ± 0.0 | 10 | 100.0 ± 0.0 | 29.0 ± 5.7 |  |
| ***ttg1-1* (L*er*)** | 1 | 10 | 99.0 ± 3.2 | 34.0 ± 8.4 | 10 | 100.0 ± 0.0 | 49.0 ± 7.4 |  |
| ***wer-1* (Col-0)** | 1 | 10 | 100.0 ± 0.0 | 39.0 ± 5.7 | 10 | 100.0 ± 0.0 | 47.0 ± 8.2 |  |
| ***gl2-1* (L*er*)** | 1 | 10 | 100.0 ± 0.0 | 56.0 ± 8.4 | 10 | 100.0 ± 0.0 | 56.0 ± 7.0 |  |
| ***try-JC* (Col-0)** | 1 | 10 | 99.0 ± 3.2 | 0.0 ± 0.0 | 10 | 100.0 ± 0.0 | 26.0 ± 7.0 |  |
| ***cpc-2* (Col-0)** | 1 | 10 | 25.0 ± 5.3 | 0.0 ± 0.0 | 10 | 58.0 ± 7.9 | 0.0 ± 0.0 |  |
| ***etc1-1* (Col-0)** | 1 | 10 | 96.0 ± 5.2 | 10.0 ± 9.4 | 10 | 100.0 ± 0.0 | 19.0 ± 8.8 |  |
| ***cpc-2 etc1-1* (Col-0)** | 1 | 11 | 13.6 ± 5.0 | 0.0 ± 0.0 | 10 | 14.0 ± 5.2 | 0.0 ± 0.0 |  |
| **Col-0** | 2 | 11 | 94.0 ± 7.0 | 0.0 ± 0.0 | 15 | 100.0 ± 0.0 | 26.4 ± 8.4 |  |
| **L*er*** | 2 | 14 | 96.9 ± 4.8 | 0.0 ± 0.0 | 11 | 100.0 ± 0.0 | 28.0 ± 6.3 |  |
| ***ttg1-1* (L*er*)** | 2 | 11 | 99.0 ± 3.2 | 28.0 ± 6.3 | 11 | 100.0 ± 0.0 | 30.0 ± 8.2 |  |
| ***wer-1* (Col-0)** | 2 | 11 | 97.0 ± 6.7 | 51.0 ± 9.9 | 13 | 100.0 ± 0.0 | 48.3 ± 9.4 |  |
| ***gl2-1* (L*er*)** | 2 | 11 | 100.0 ± 0.0 | 56.0 ± 5.2 | 14 | 100.0 ± 0.0 | 50.8 ± 9.5 |  |
| ***try-JC* (Col-0)** | 2 | 11 | 95.0 ± 5.3 | 0.0 ± 0.0 | 14 | 100.0 ± 0.0 | 28.5 ± 8.0 |  |
| ***cpc-2* (Col-0)** | 2 | 11 | 43.0 ± 6.7 | 0.0 ± 0.0 | 11 | 54.0 ± 7.0 | 0.0 ± 0.0 |  |
| ***etc1-1* (Col-0)** | 2 | 11 | 86.0 ± 7.0 | 0.0 ± 0.0 | 11 | 100.0 ± 0.0 | 23.0 ± 4.8 |  |
| ***cpc-2 etc1-1* (Col-0)** | 2 | 13 | 15.0 ± 5.2 | 0.0 ± 0.0 | 11 | 16.0 ± 5.2 | 0.0 ± 0.0 |  |

Exp.: experiment, n: number of analyzed seedlings
