# Supplementary material for: Sub-epidermal Expression of ENHANCER OF TRIPTYCHON AND CAPRICE1 and Its Role in Root Hair Formation Upon Pi Starvation
Source: Front Plant Sci. 2018 Sep 27;9:1411. doi: 10.3389/fpls.2018.01411 (PMC6171471; doi:10.3389/fpls.2018.01411)
Supplement: Supplementary file 3 [file Table_3.docx]

**Table S3**: Significance test results (*P*-values from Wilcoxon test, unpaired) of the difference between the Pi+ and Pi- condition for file-specific root hair cell percentages of each genotype (see also Figure 1 and Table S2).

| **Genotype** |  | ***P*-value** | |
| --- | --- | --- | --- |
|  | **Experiment** | **H-file** | **N-file** |
| **Col-0** | 1 | 0.0767 | 4.7E-05 |
| **L*er*** | 1 | 0.0143 | 4.1E-05 |
| ***ttg1-1* (L*er*)** | 1 | 0.3681 | 0.0020 |
| ***wer-1* (Col-0)** | 1 | NA | 0.0286 |
| ***gl2-1* (L*er*)** | 1 | NA | 0.8995 |
| ***try-JC* (Col-0)** | 1 | 0.3681 | 5.1E-05 |
| ***cpc-2* (Col-0)** | 1 | 1.3E-04 | NA |
| ***etc1-1* (Col-0)** | 1 | 0.0336 | 0.0642 |
| ***cpc-2 etc1-1* (Col-0)** | 1 | 0.9002 | NA |
| **Col-0** | 2 | 0.0043 | 1.4E-05 |
| **L*er*** | 2 | 0.0657 | 7.8E-06 |
| ***ttg1-1* (L*er*)** | 2 | 0.3681 | 0.5924 |
| ***wer-1* (Col-0)** | 2 | 0.1286 | 0.5401 |
| ***gl2-1* (L*er*)** | 2 | NA | 0.1848 |
| ***try-JC* (Col-0)** | 2 | 0.0055 | 2.4E-05 |
| ***cpc-2* (Col-0)** | 2 | 0.0030 | NA |
| ***etc1-1* (Col-0)** | 2 | 1.9E-04 | 4.0E-05 |
| ***cpc-2 etc1-1* (Col-0)** | 2 | 0.6744 | NA |
