# Supplementary material for: Sub-epidermal Expression of ENHANCER OF TRIPTYCHON AND CAPRICE1 and Its Role in Root Hair Formation Upon Pi Starvation
Source: Front Plant Sci. 2018 Sep 27;9:1411. doi: 10.3389/fpls.2018.01411 (PMC6171471; doi:10.3389/fpls.2018.01411)
Supplement: Supplementary file 4 [file Table_4.docx]

**Table S4**: Significance test results (*P*-values from Wilcoxon test, unpaired) of the difference between each mutant and its corresponding wild type for file-specific root hair cell percentage at both Pi conditions (see also Figure 1 and Table S2).

|  |  | ***P*-value** | | | |
| --- | --- | --- | --- | --- | --- |
| **Genotype** |  | **Phosphate sufficient (Pi+)** | | **Phosphate deficient (Pi-)** | |
|  | **Experiment** | **H-file** | **N-file** | **H-file** | **N-file** |
| ***ttg1-1* (L*er*)** | 1 | 0.0593 | 5.3E-05 | NA | 1.7E-04 |
| ***wer-1* (Col-0)** | 1 | 0.0767 | 7.5E-05 | NA | 1.5E-04 |
| ***gl2-1* (L*er*)** | 1 | 0.0143 | 4.8E-05 | NA | 1.1E-04 |
| ***try -JC* (Col-0)** | 1 | 0.3006 | 0.1675 | NA | 0.0485 |
| ***cpc-2* (Col-0)** | 1 | 1.0E-04 | 0.1675 | 5.5E-05 | 2.4E-05 |
| ***etc1-1* (Col-0)** | 1 | 0.6809 | 0.0429 | NA | 0.0020 |
| ***cpc-2 etc1-1* (Col-0)** | 1 | 6.2E-05 | 0.1463 | 4.6E-05 | 2.4E-05 |
| ***ttg1-1* (L*er*)** | 2 | 0.2596 | 7.8E-06 | NA | 0.5924 |
| ***wer-1* (Col-0)** | 2 | 0.2437 | 5.1E-05 | NA | 1.9E-05 |
| ***gl2-1* (L*er*)** | 2 | 0.0657 | 7.5E-06 | NA | 1.5E-04 |
| ***try -JC* (Col-0)** | 2 | 0.8646 | NA | NA | 0.7308 |
| ***cpc-2* (Col-0)** | 2 | 1.3E-04 | NA | 3.9E-06 | 1.4E-05 |
| ***etc1-1* (Col-0)** | 2 | 0.0271 | NA | NA | 0.1377 |
| ***cpc-2 etc1-1* (Col-0)** | 2 | 5.4E-05 | NA | 4.2E-06 | 1.4E-05 |
