# Supplementary material for: Sub-epidermal Expression of ENHANCER OF TRIPTYCHON AND CAPRICE1 and Its Role in Root Hair Formation Upon Pi Starvation
Source: Front Plant Sci. 2018 Sep 27;9:1411. doi: 10.3389/fpls.2018.01411 (PMC6171471; doi:10.3389/fpls.2018.01411)
Supplement: Supplementary file 5 [file Table_5.docx]

**Table S5** Qualitative analysis of a set of *ETC1* promoter deletion constructs transformed into *cpc-2 etc1-1*. Given is the number of BASTA resistant T1 seedlings per construct and the number of these seedlings with root hairs as compared to *cpc-2 etc1-1* mutants. Two independent lines for each construct were quantitatively analyzed in the T_2_ generation.

|  | BASTA resistant | with root hairs |
| --- | --- | --- |
| *ProETC1*^-1921^:ETC1 (*cpc-2 etc1-1*) | 20 | 8 |
| *ProETC1*^-1676^:ETC1 (*cpc-2 etc1-1*) | 26 | 9 |
| *ProETC1*^-1371^:ETC1 (*cpc-2 etc1-1*) | 8 | 2 |
| *ProETC1*^-1183^:ETC1 (*cpc-2 etc1-1*) | 41 | 7 |
| *ProETC1*^-932^:ETC1 (*cpc-2 etc1-1*) | 40 | 10 |
| *ProETC1*^-595^:ETC1 (*cpc-2 etc1-1*) | 40 | 0 |
| *ProETC1*^-400^:ETC1 (*cpc-2 etc1-1*) | 40 | 0 |
