# Supplementary material for: Sub-epidermal Expression of ENHANCER OF TRIPTYCHON AND CAPRICE1 and Its Role in Root Hair Formation Upon Pi Starvation
Source: Front Plant Sci. 2018 Sep 27;9:1411. doi: 10.3389/fpls.2018.01411 (PMC6171471; doi:10.3389/fpls.2018.01411)
Supplement: Supplementary file 6 [file Table_6.docx]

**Table S6. Pi dependent rescue ability of ETC1 promoter fragments.** *ETC1* promoter rescue analysis and effect of Pi availability on relative root hair cell numbers in the RHC (see Figure 1). Numbers in superscript indicate the distance to the ETC1 start codon at which the respective construct begins. 7- day old *Arabidopsis thaliana* wild-type and mutant seedlings were analyzed at each condition. Values represent the mean percentages of root hair cells in H- or N-files in the patterning zone of primary roots of ten plants, respectively. (mean percentage ± SD). (For statistics see Tables S7-9).

|  | **Root hair cell [%]** | | | |
| --- | --- | --- | --- | --- |
|  | **Phosphate sufficient (Pi+)** | | **Phosphate deficient (Pi-)** | |
| **Genotype** | **H-file** | **N-file** | **H-file** | **N-file** |
| ***cpc-2 etc1-1*** | 7.0 ± 6.7 | 0.0 ± 0.0 | 9.0 ± 7.4 | 0.0 ± 0.0 |
| ***cpc-2*** | 33.0 ± 8.2 | 0.0 ± 0.0 | 55.0 ± 7.1 | 0.0 ± 0.0 |
|  |  |  |  |  |
| **Pro*ETC1^-1371^*: YFP-ETC1**  **(*cpc-2 etc1-1*) line I** | 36.0 ± 9.7 | 0.0 ± 0.0 | 63.0 ± 9.5 | 2.0 ± 4.2 |
| **Pro*ETC1^-1371^*: YFP-ETC1**  **(*cpc-2 etc1-1*) line II** | 42.0 ± 4.2 | 0.0 ± 0.0 | 65.0 ± 5.3 | 3.0 ± 4.8 |
|  |  |  |  |  |
| **Pro*ETC^-1921^*: ETC1**  **(*cpc-2 etc1-1*) line 1** | 38.0 ± 9.2 | 0.0 ± 0.0 | 61.0 ± 9.9 | 3.0 ± 4.8 |
| **Pro*ETC^c-1921^*: ETC1**  **(*cpc-2 etc1-1*) line 8** | 36.0 ± 9.7 | 0.0 ± 0.0 | 64.4 ± 8.8 | 2.2 ± 4.4 |
| **Pro*ETC1^-1676^*: ETC1**  **(*cpc-2 etc1-1*) line 2** | 43.0 ± 23.1 | 0.0 ± 0.0 | 85 ± 16.5 | 0.0 ± 0.0 |
| **Pro*ETC1^-1676^*: ETC1**  **(*cpc-2 etc1-1*) line 1** | 20.0 ± 18.9 | 0.0 ± 0.0 | 58.6 ± 12.1 | 0.0 ± 0.0 |
| **Pro*ETC1^-1371^*: ETC1**  **(*cpc-2 etc1-1*) line 1** | 46.0 ± 17.8 | 0.0 ± 0.0 | 89.0 ± 14.5 | 1.0 ± 3.2 |
| **Pro*ETC1^-1371^*: ETC1**  **(*cpc-2 etc1-1*) line 4** | 30.0 ± 8.2 | 0.0 ± 0.0 | 62.0 ± 9.2 | 0.0 ± 0.0 |
| **Pro*ETC1^-1183^*: ETC1**  **(*cpc-2 etc1-1*) line 2** | 36.0 ± 13.5 | 0.0 ± 0.0 | 50.0 ± 8.2 | 0.0 ± 0.0 |
| **Pro*ETC1^-1183^*: ETC1**  **(*cpc-2 etc1-1*) line 17** | 21.0 ± 22.3 | 0.0 ± 0.0 | 65.0 ± 5.3 | 2.0 ± 4.2 |
| **Pro*ETC1^-932^*: ETC1**  **(*cpc-2 etc1-1*) line 28** | 34.0 ± 5.2 | 0.0 ± 0.0 | 73.3 ± 12.2 | 0.0 ± 0.0 |
| **Pro*ETC1^-932^*: ETC1**  **(*cpc-2 etc1-1*) line 19** | 46.0 ± 7.0 | 0.0 ± 0.0 | 61.0 ± 12.9 | 0.0 ± 0.0 |
| **Pro*ETC1^-595^*: ETC1**  **(*cpc-2 etc1-1*) line 26** | 7.0 ± 6.7 | 0.0 ± 0.0 | 11.0 ± 5.7 | 0.0 ± 0.0 |
| **Pro*ETC1^-595^*: ETC1**  **(*cpc-2 etc1-1*) line 5** | 6.0 ± 7.0 | 0.0 ± 0.0 | 8.8 ± 6.4 | 0.0 ± 0.0 |
| **Pro*ETC1^-400^*: ETC1**  **(*cpc-2 etc1-1*) line 39** | 8.0 ± 6.3 | 0.0 ± 0.0 | 11.0 ± 7.4 | 0.0 ± 0.0 |
| **Pro*ETC1^-400^*: ETC1**  **(*cpc-2 etc1-1*) line 12** | 8.0 ± 7.9 | 0.0 ± 0.0 | 7.0 ± 6.7 | 0.0 ± 0.0 |
| **Pro*ETC1*^PHR1mut^: ETC1**  **(*cpc-2 etc1-1*) line 3** | 39.0 ± 7.4 | 0.0 ± 0.0 | 57 ± 9.5 | 1.0 ± 3.2 |
