# Supplementary material for: Sub-epidermal Expression of ENHANCER OF TRIPTYCHON AND CAPRICE1 and Its Role in Root Hair Formation Upon Pi Starvation
Source: Front Plant Sci. 2018 Sep 27;9:1411. doi: 10.3389/fpls.2018.01411 (PMC6171471; doi:10.3389/fpls.2018.01411)
Supplement: Supplementary file 8 [file Table_8.docx]

**Table S8 Pi dependent rescue ability of ETC1 promoter fragments – statistics**: Significance test results (*P*-values from Wilcoxon test, unpaired) of the differences between each genotype and the *cpc-2* mutant for file-specific root hair cell percentage at both Pi conditions (Table S6).

|  | ***P*-value** | | | |
| --- | --- | --- | --- | --- |
| **Genotype** | **Phosphate sufficient (Pi+)** | | **Phosphate deficient (Pi-)** | |
|  | **H-file** | **N-file** | **H-file** | **N-file** |
| ***cpc-2 etc1-1*** | 0.0001 | NA | 0.0001 | NA |
|  |  |  |  |  |
| **Pro*ETC1^-1371^*:YFP-ETC1**  **(*cpc-2 etc1-1*) line I** | 0.3366 | NA | 0.0541 | 0.1675 |
| **Pro*ETC1^-1371^*:YFP-ETC1**  **(*cpc-2 etc1-1*) line II** | 0.0076 | NA | 0.0055 | 0.0767 |
|  |  |  |  |  |
| **Pro*ETC^c-1921^*: ETC1**  **(*cpc-2 etc1-1*) line 1** | 0.2424 | NA | 0.1548 | 0.0767 |
| **Pro*ETC1^-1921^*: ETC1**  **(*cpc-2 etc1-1*) line 8** | 0.4624 | NA | 0.0247 | 0.1451 |
| **Pro*ETC1^-1676^*: ETC1**  **(*cpc-2 etc1-1*) line 2** | 0.0356 | NA | 0.0006 | NA |
| **Pro*ETC1^-1676^*: ETC1**  **(*cpc-2 etc1-1*) line 1** | 0.1198 | NA | 0.4971 | NA |
| **Pro*ETC1^-1371^*: ETC1**  **(*cpc-2 etc1-1*) line 1** | 0.0093 | NA | 0.0002 | 0.3681 |
| **Pro*ETC1^-1371^*: ETC1**  **(*cpc-2 etc1-1*) line 4** | 0.5127 | NA | 0.0747 | NA |
| **Pro*ETC1^-1183^*: ETC1**  **(*cpc-2 etc1-1*) line 2** | 0.7763 | NA | 0.2178 | NA |
| **Pro*ETC1^-1183^*: ETC1**  **(*cpc-2 etc1-1*) line 17** | 0.5310 | NA | 0.0055 | 0.1675 |
| **Pro*ETC1^-932^*: ETC1**  **(*cpc-2 etc1-1*) line 28** | 0.6331 | NA | 0.0040 | NA |
| **Pro*ETC1^-932^*: ETC1**  **(*cpc-2 etc1-1*) line 19** | 0.0031 | NA | 0.1568 | NA |
| **Pro*ETC1^-595^*: ETC1**  **(*cpc-2 etc1-1*) line 26** | 0.0001 | NA | 0.0001 | NA |
| **Pro*ETC1^-595^*: ETC1**  **(*cpc-2 etc1-1*) line 5** | 0.0001 | NA | 0.0003 | NA |
| **Pro*ETC1^-400^*: ETC1**  **(*cpc-2 etc1-1*) line 39** | 0.0001 | NA | 0.0001 | NA |
| **Pro*ETC1^-400^*: ETC1**  **(*cpc-2 etc1-1*) line 12** | 0.0002 | NA | 0.0001 | NA |
| **Pro*ETC1*^PHR1mut^: ETC1**  **(*cpc-2 etc1-1*) line 3** | 0.0956 | NA | 0.5421 | 0.3681 |
