# Supplementary material for: Sub-epidermal Expression of ENHANCER OF TRIPTYCHON AND CAPRICE1 and Its Role in Root Hair Formation Upon Pi Starvation
Source: Front Plant Sci. 2018 Sep 27;9:1411. doi: 10.3389/fpls.2018.01411 (PMC6171471; doi:10.3389/fpls.2018.01411)
Supplement: Supplementary file 9 [file Table_9.docx]

**Table S9 Pi dependent rescue ability of ETC1 promoter fragments – statistic**: Significance test results (*P*-values from Wilcoxon test, unpaired) between the Pi+ and Pi- condition for file-specific root hair cell percentages of each genotype (Table S6).

| **Genotype** | ***P*-value** | |
| --- | --- | --- |
|  | **H-file** | **N-file** |
| ***cpc-2 etc1-1*** | 0.5616 | NA |
| ***cpc-2*** | 0.0003 | NA |
|  |  |  |
| **Pro*ETC1^-1371^*:YFP-ETC1**  **(*cpc-2 etc1-1*) line I** | 0.0002 | 0.1675 |
| **Pro*ETC1^-1371^*:YFP-ETC1**  **(*cpc-2 etc1-1*) line II** | 8.5E-05 | 0.0767 |
|  |  |  |
| **Pro*ETC^c-1921^*: ETC1**  **(*cpc-2 etc1-1*) line 1** | 0.0005 | 0.0767 |
| **Pro*ETC1^-1921^*: ETC1**  **(*cpc-2 etc1-1*) line 8** | 0.0003 | 0.1451 |
| **Pro*ETC1^-1676^*: ETC1**  **(*cpc-2 etc1-1*) line 2** | 0.0003 | NA |
| **Pro*ETC1^-1676^*: ETC1**  **(*cpc-2 etc1-1*) line 1** | 0.0015 | NA |
| **Pro*ETC1^-1371^*: ETC1**  **(*cpc-2 etc1-1*) line 1** | 0.0001 | 0.3681 |
| **Pro*ETC1^-1371^*: ETC1**  **(*cpc-2 etc1-1*) line 4** | 0.0001 | NA |
| **Pro*ETC1^-1183^*: ETC1**  **(*cpc-2 etc1-1*) line 2** | 0.0222 | NA |
| **Pro*ETC1^-1183^*: ETC1**  **(*cpc-2 etc1-1*) line 17** | 0.0001 | 0.1675 |
| **Pro*ETC1^-932^*: ETC1**  **(*cpc-2 etc1-1*) line 28** | 0.0002 | NA |
| **Pro*ETC1^-932^*: ETC1**  **(*cpc-2 etc1-1*) line 19** | 0.0109 | NA |
| **Pro*ETC1^-595^*: ETC1**  **(*cpc-2 etc1-1*) line 26** | 0.1673 | NA |
| **Pro*ETC1^-595^*: ETC1**  **(*cpc-2 etc1-1*) line 5** | 0.3764 | NA |
| **Pro*ETC1^-400^*: ETC1**  **(*cpc-2 etc1-1*) line 39** | 0.3561 | NA |
| **Pro*ETC1^-400^*: ETC1**  **(*cpc-2 etc1-1*) line 12** | 0.8370 | NA |
| **Pro*ETC1*^PHR1mut^: ETC1**  **(*cpc-2 etc1-1*) line 3** | 0.0011 | 0.3681 |
