# Supplementary material for: Sub-epidermal Expression of ENHANCER OF TRIPTYCHON AND CAPRICE1 and Its Role in Root Hair Formation Upon Pi Starvation
Source: Front Plant Sci. 2018 Sep 27;9:1411. doi: 10.3389/fpls.2018.01411 (PMC6171471; doi:10.3389/fpls.2018.01411)
Supplement: Supplementary file 10 [file Table_10.docx]

**Table S10:** cis-regulatory elements in the region -932 to -595 of *ETC1* promoter as predicted by PLACE. Only *A. thaliana* motifs were considered

___________________________________________________________________________

| **Cis-element** | **Sequence** | **Number** | **Function** |
| --- | --- | --- | --- |
| ABRELATERD1 | ACGTG | 1 | ABRE-like sequence (from -199 to -195) required for etiolation-induced expression of erd1 |
| ABRERATCAL | MACGYGB | 1 | "ABRE-related sequence" identified  in the upstream regions of 162 Ca(2+)-responsive upregulated genes |
| ACGTATERD1 | ACGT | 3 | ACGT sequence required for etiolation-induced expression of erd1 |
| ARR1AT | NGATT | 4 | ARR1-binding element |
| ASF1MOTIFCAMV | TGACG | 1 | ASF-1 binding site |
| CACGTGMOTIF | CACGTG | 1 | G-box; Binding site of Arabidopsis GBF4 |
| CARGCW8GAT | CWWWWWWWWG | 1 | A variant of CArG motif |
| GADOWNAT | ACGTGTC | 1 | Sequence present in 24 genes in the GA-down regulated d1 cluster(106 genes) found in Arabidopsis seed germination |
| GAREAT | TAACAAR | 1 | GARE (GA-responsive element) |
| GATA box | GATA | 4 | GATA box |
| GT1CONSENSUS | GRWAAW | 1 | Consensus GT-1 binding site in many light-regulated genes |
| IBOXCORE | GATAA | 1 | I-box; Conserved sequence upstream of light-regulated genes |
| MYB2CONSENSUSAT | YAACKG | 1 | MYB recognition site |
| MYCCONSENSUSAT | CANNTG | 2 | MYC recognition site |
| P1BS | GNATATNC | 1 | PHR1-binding sequence |
| POLASIG1 | AATAAA | 3 | PolyA signal |
| RAV1AAT | CAACA | 1 | Binding consensus sequence of Arabidopsis transcription factor, RAV1 |
| WBOXATNPR1 | TTGAC | 1 | WRKY binding site |

N stands for any base, Y represents any [pyrimidine](http://en.wikipedia.org/wiki/Pyrimidine), W = A T
