# Supplementary material for: Sub-epidermal Expression of ENHANCER OF TRIPTYCHON AND CAPRICE1 and Its Role in Root Hair Formation Upon Pi Starvation
Source: Front Plant Sci. 2018 Sep 27;9:1411. doi: 10.3389/fpls.2018.01411 (PMC6171471; doi:10.3389/fpls.2018.01411)
Supplement: Supplementary file 12 [file Table_12.docx]

**Table S12 Rescue experiment using Pro*SCR*:*YFP-ETC1*.** Pro*SCR*:*YFP-ETC1* rescues the *cpc-2 etc1-1* mutant. Values represent the mean percentages of root hair cells in an H- or N-file in the RHC (see Figure 1), respectively (mean percentage ± SD). Primary roots of seven to twenty 7- day old seedlings were analyzed.

|  |  |  |  |  |
| --- | --- | --- | --- | --- |
|  |  |  | **Phosphate sufficient (Pi+)** | |
| **Experiment** | **Genotype** | **Line ID** | **H-file** | **N-file** |
| **A^#^** | **Pro*SCR*:*YFP-ETC1* (*cpc-2 etc1-1*)** | I | 61.8 ± 21.5 (10) | 0.0 ± 0.0 (10) |
| **A^#^** | **Pro*SCR*:*YFP-ETC1* (*cpc-2 etc1-1*)** | 11 | 85.9 ± 11.4 (8) | 1.8 ± 3.7 (8) |
| **A^#^** | **Pro*SCR*:*YFP-ETC1* (*cpc-2 etc1-1*)** | 12 | 61.1 ± 26.6 (5) | NA |
| **A^#^** | **Col-0** | - | 100.0 ± 0.0 (7) | 5.7 ± 7.8 (7) |
| **B** | **Pro*SCR*:*YFP-ETC1* (*cpc-2 etc1-1*)** | I | 91.4 ± 13.6 (15) | 0.0 ± 0.0 (15) |
| **B** | **Col-0** | - | 100.0 ± 0.0 (10) | 12.5 ± 31.7 (10) |
| **C** | **Pro*SCR*:*YFP-ETC1* (*cpc-2 etc1-1*)** | I | 69.8 ± 23.3 (10) | 2.0 ± 6.3 (10) |
| **D** | **Pro*SCR*:*YFP-ETC1* (*cpc-2 etc1-1*)** | I | 75.0 ± 12.0 (20) | 2.8 ± 6.4 (20) |

#: in this experiment *cpc-2 etc1-1* was qualitatively analyzed in parallel and almost no root hairs were found along the whole root (comparable to the results documented in Figure 1). In brackets: number of seedlings analyzed. NA: data not acquired.
