# Supplementary material for: Sub-epidermal Expression of ENHANCER OF TRIPTYCHON AND CAPRICE1 and Its Role in Root Hair Formation Upon Pi Starvation
Source: Front Plant Sci. 2018 Sep 27;9:1411. doi: 10.3389/fpls.2018.01411 (PMC6171471; doi:10.3389/fpls.2018.01411)
Supplement: Supplementary file 13 [file Table_13.docx]

Table S13: List of primers

| **Primer name** | **Primer sequence** | **Source** |
| --- | --- | --- |
| GW-pETC1 F | GGGGACAAGTTTGTACAAAAAAGCAGGCTTCGAGAAAAGACTTTGAACTTGCAC | This study |
| GW-pETC1 R | GGGGACCACTTTGTACAAGAAAGCTGGGTCCTTCAGAAGAAGAGGCAAC | This study |
| GW-pETC1 -1676 F | GGGGACAAGTTTGTACAAAAAAGCAGGCTTCGATTCCAATATCTAGGCTG | This study |
| GW-pETC1 -1371 F | GGGGACAAGTTTGTACAAAAAAGCAGGCTTCGGTTGAGATATCCTTAGC | This study |
| GW-pETC1 -1183 F | GGGGACAAGTTTGTACAAAAAAGCAGGCTTCTGGGTTCTCTATAGTCTC | This study |
| GW-*pETC1 -932* F | GGGGACAAGTTTGTACAAAAAAGCAGGCTTCGTGAAGGCGTCATGAATC | This study |
| GW-*pETC1 -595* | GGGGACAAGTTTGTACAAAAAAGCAGGCTTCGGACTTCATAAATGATAATC | This study |
| GW-*pETC1 -400* | GGGGACAAGTTTGTACAAAAAAGCAGGCTTCGACAAGAACATGACGGAGC | This study |
| GW-ETC1 cDNA F | GGGGACAAGTTTGTACAAAAAAGCAGGCTTCATCATTCTCTCTTCCTCTCTG | This study |
| GW-ETC1 CDS R | GGGGACCACTTTGTACAAGAAAGCTGGGTC TACATATCCACAAATTATT | This study |
| Asc1-pETC1 F | GGCGCGCC GAGAAAAGACTTTGAACTTGCAC | This study |
| Xho1-pETC1 R | CTCGAG CTTCAGAAGAAGAGGCAAC | This study |
| phr1mut- F | GGAGGAATAAAGGAAAAAAAATCGACGTTAAATTAATTTTATATTC | This study |
| phr1mut- R | GAATATAAAATTAATTTAACGTCGATTTTTTTTCCTTTATTCCTCC | This study |
| Asc1-pSCR F | GGCGCGCCAGATTGTGATCCTCTGCAAC | Rishmawi et al., 2014 |
| Xho1-pSCR R | CTCGAGGGAGATTGAAGGGTTGTTGG | Rishmawi et al., 2014 |
| ANS89SpeI-HA-GUS-s | GAGAGAACTAGTATGTACCCATACGATGTTCCAGATTACGCTATGAGGGAATTCATGTTACGTC | This study |
| ANS90SpeI-GUS-as | GAGAGAACTAGTTCAGTCGACCCCGGGGCCCATGGGTTGTTTGCCTCCCTGC | This study |
